# Reviewers

**DOI:** 10.1093/jncics/pkae055

**Published:** 2024-09-23

In celebration of Peer Review Week (September 23–27, 2024) we wish to thank the following reviewers for their generous dedication of time in evaluating manuscripts submitted to *JNCI Cancer Spectrum* during the past year. Those who were our top reviewers, turning in 3 or more reviews, are indicated with asterisks.

Yasemin Adali*

Prajakta Adsul

Ilir Agalliu

Piyush K. Agarwal

Jaeil Ahn

David Akhavan

Nada Al-Antary

Trisha Amboree

Saber Amin

Ming-Wen An

Hannah Arem

Volker Arndt

Elizabeth Arthur

Nikita Baclig

Ana Barac

Jorge Barriuso*

Melissa Beauchemin

Tanios Bekaii-Saab

Keith Bellizzi

Al Benson III

Nathan Berger

Marianne Berwick

Ana Best

Traci Bethea*

Douglas Blayney

Jaine Blayney

Paolo Boffetta

Jeffrey Bogart

Quiera Booker

David Boulate

Dana Bovbjerg

Sarah Bowen

Angela Bradbury

Natalie Bradford

Cathy Bradley

Kristen Brantley

Christa Braun-Inglis

Karlynn BrintzenhofeSzoc

Austin Brown

Michael Brundage

Paul Bunn Jr

Ying Cao

Enrico Capobianco

Marcelo Cerullo

Young Chandler

David Chang

George Chang

Devon Check

Zhengyi Chen

En Cheng

Rowan Chlebowski

Alfredo Jr. Chua

Timothy Church

Romain Cohen

Ciara Conduit

James Cone

Eileen Connolly

Katherine Crew

Jennifer Croswell

Bhudev Das

Claire de la Calle

Suzette Delaloge

Manali Desai

Michaela Dinan*

Andreas Dinkel

Kevin Dobbin

Yun Du*

Kevin Elias*

Christen Elledge

Katherine Enright

Adam Esbenshade

Michael S. Ewer

Jonine Figueroa

Gunnar Folprecht

Lorenzo Fornaro

Sophie D. Fossa

Valentyn Fournier

Zachary Frosch

Bernard Fuemmeler

Emily Gallagher

Lisa Gallicchio

Gregory Gan

Patricia Ganz

Elizabeth Garrett-Mayer

Stephanie George

Sophia George

Daniel Geynisman

Karthik Ghosh

Peter Gibbs

Edward Giovannucci

Bonnie Gould Rothberg

Ilana Graetz

Marjolein Greuter

Cary Gross

Axel Grothey

X. Mona Guo

Russell Hales

Balazs Halmos

Summer Han

Ashley Henneghan

Marc Hoffmann

Julian Hong

Chi-Chen Hong

Chunsong Hu

Wei-Ting Hwang

Lynn Ibekwe

Manami Inoue

Joseph O. Jacobson

Michelle Janelsins

Stephanie Jarosek

Ismail Jatoi

Megan Jeon

Krishan Jethwa

Bruce Johnson

Rena Jones

Himanshu Joshi

John Kairalla

Suneel Kamath

Michael Kammer

Xiaozheng Kang

Lawrence Kasherman

Aaron Katz

Nancy Keating

Kayleigh Elizabeth Keith

Erin Kent

Hiba Khan

Kim Kimminau

Amber Kleckner

Zdenek Kleibl

Jennifer Klemp

Boguslawa Koczwara

Shunsuke Koga

Manish Kohli

Hope Krebill

Tzy-Mey Kuo

Miranda Lam

Wendy Landier

Marie Lange

Theodore Lawrence

Michael Leapman

Heather Leeper

William Letsou

John Liao

Jennifer Ligibel

Frank Lin

Simon Lo

Yujia Lu

Philip Lupo

Jeanne Mandelblatt

Maria-Victoria Mateos

Paola Mattiolo

Deborah Mayer

Erica Mayer

Gina Mazza*

Marjorie McCullough

Valentin H. Meissner

Michelle Melis

Christine Miaskowski

Daniel Michaeli

Rachel Michaelson

Tamara Miller

Aaron Mitchell*

Zahi Mitri

Rahma Mkuu

Justin Xavier Moore

Motomi Mori

Carolyn Muller

Steven Narod*

Sarah Nash

Manan Nayak

Sarah Nechuta

Larissa Nekhlyudov

Mytien Nguyen

Zhenhui Nie

Leticia Nogueira

Izumi Okado

Ahmad Ozair

I-Wen Pan*

Lani Park

Electra Paskett

Debra Patt

Christine L. Phillips

Paulo Pinheiro

Charles Porter

Melanie Potiaumpai

Shelley Potter

Stephanie Pugh

Jin Qin

Ryan Rader

Ramya Ramaswami

Cody Ramin

Max Carlos Ramírez-Soto

Huma Rana

Maija Reblin

Krishna Reddy

Kelly Rentscher

Lisa Richardson

Mya Roberson

Eduardo José Fernández Rodríguez

B. R. Rosser

Lawrence Rubinstein

Roberto Salgado

John Salsman

Barbara Saltzman

Taneisha Scheuermann

John Schiller

Mario Schootman

Jeremy Schraw

Tyler Seibert

Jason Semprini

Shomik Sengupta

Diego Serraino

Urvi Shah

Salma Shariff-Marco

Priyanka Sharma

Dylan Sherry

Jay Shiao

David Shibata*

Peter Shields

Naoko Simonds

Logan Spector

Srikala Sridhar

Susan Steck

Nicole Stout

Leslie Sude

Zhuoxin Sun

Karey Sutton

Emanuela Taioli

Rulla Tamimi

Ray Tan*

Ian Tannock

Kekoa Taparra

Sarah Temkin

Joel Tepper

Bridgette Thom

Caroline Thompson

Jeffrey Tice

Meghan Tipre

Angelina Tjokrowidjaja

Julia Trosman

Nishant Uppal

Saad Usmani

Susan Vadaparampil

Jonathon Vallejo

Mieke Van Hemelrijck

Marina van Leeuwen*

Robin Vanderpool

Rebecca Venchiarutti

Matthew Vernon

Samuel Volchenboum

Colton Walker

Kristin Wallace

Peng Wang

Samuel Washington III

Rachel Webster

Whitney Welch Morelli

Nicholas Wilcox

James Willey

AnnaLynn Williams

Jan Wohlfahrt

F. Lennie Wong

Yu-Ning Wong

Marie Wood*

Alice Yan

Juan Yang

Elizabeth Yanik

Song Yao

Saunjoo Yoon

Takeshi Yuasa

Alison Zhang

Houyu Zhao

Eric Zhou

Jeanette Ziegenfuss

Argyrios Ziogas

Jamie Zoellner

